# Random-Effects, Fixed-Effects and the within-between Specification for Clustered Data in Observational Health Studies: A Simulation Study

**DOI:** 10.1371/journal.pone.0110257

**Published:** 2014-10-24

**Authors:** Joseph L. Dieleman, Tara Templin

**Affiliations:** Institute for Health Metrics and Evaluation, University of Washington, Seattle, Washington, United States of America; University of Westminster, United Kingdom

## Abstract

**Background:**

When unaccounted-for group-level characteristics affect an outcome variable, traditional linear regression is inefficient and can be biased. The random- and fixed-effects estimators (RE and FE, respectively) are two competing methods that address these problems. While each estimator controls for otherwise unaccounted-for effects, the two estimators require different assumptions. Health researchers tend to favor RE estimation, while researchers from some other disciplines tend to favor FE estimation. In addition to RE and FE, an alternative method called within-between (WB) was suggested by Mundlak in 1978, although is utilized infrequently.

**Methods:**

We conduct a simulation study to compare RE, FE, and WB estimation across 16,200 scenarios. The scenarios vary in the number of groups, the size of the groups, within-group variation, goodness-of-fit of the model, and the degree to which the model is correctly specified. Estimator preference is determined by lowest mean squared error of the estimated marginal effect and root mean squared error of fitted values.

**Results:**

Although there are scenarios when each estimator is most appropriate, the cases in which traditional RE estimation is preferred are less common. In finite samples, the WB approach outperforms both traditional estimators. The Hausman test guides the practitioner to the estimator with the smallest absolute error only 61% of the time, and in many sample sizes simply applying the WB approach produces smaller absolute errors than following the suggestion of the test.

**Conclusions:**

Specification and estimation should be carefully considered and ultimately guided by the objective of the analysis and characteristics of the data. The WB approach has been underutilized, particularly for inference on marginal effects in small samples. Blindly applying any estimator can lead to bias, inefficiency, and flawed inference.

## Introduction

Observational health studies frequently deal with grouped or clustered data. When observations are clustered into groups, common group-level characteristics can affect outcomes. If *all* of these unique characteristics are observed and measured, it would be possible to include them in a model, although in most cases this is unrealistic. Health facilities in a single geographic region may share budgets, guiding policies, attitudes towards treatment, populations, disease patterns, and constraints on supplies. If multiple facilities from multiple regions are considered in a single analysis, facilities will implicitly be clustered by region. Although these group-level commonalities affect the facilities' ability to supply services, it would be unrealistic to measure and include all of them in a model. However, not addressing the unifying group-level characteristics violates assumptions needed to prevent bias in many regressions. Fortunately, there are two relatively common methods for improving the estimation of clustered data: random- and fixed-effects estimation (RE and FE, respectively) [1]. Applying these methods can eliminate bias and improve efficiency.

The versatility of RE and FE has inspired a great deal of research and commentaries addressing both theory and practice. These works encompass the health sciences [2]–[6], social sciences [7]–[10], and econometric theory [11]–[12]. Many prior empirical studies have reviewed various specifications of RE and FE for analyzing clustered data [13]–[17]. Other studies have compared FE and RE to generalized estimating equations (GEE) [18]–[19]. Although the theory supporting the RE and FE estimators is well established, there remains little consensus across disciplines about when each is most appropriate. Debate regarding which estimator to use persists within the health sciences, even though RE, FE, and WB have been explored theoretically and using simulation [20]–[25].

**Figure 1:**
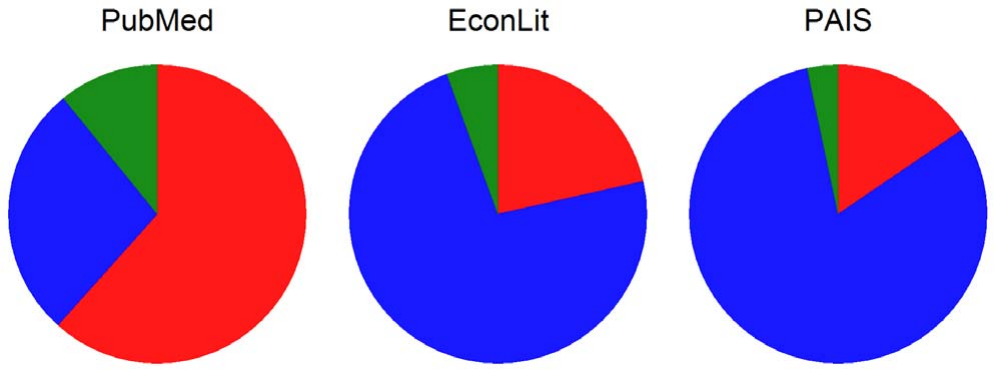
Prevalence of random- and fixed-effects in health, economics, and political science literature. Each archive was searched for the terms “random effects” or “random effect” and “fixed effects” or “fixed effect” present in abstracts. Papers that also used the term “meta” in the abstract were not included in to avoid including meta-analyses which is a very specific use of RE and FE estimation. PubMed is a database that archives life science and biomedical abstracts and references, primarily drawn from the MEDLINE database. EconLit is also an archiving database, published by the American Economic Association, which focuses on economics literature. PAIS is the Public Affairs Information Service International database, which archives references focusing on public affairs.

Figure 1 shows that health researchers have disproportionally preferred RE estimation relative to researchers in some social sciences [26]–[28]. There may be a plausible reason for this difference. Health researchers often work in the framework of a randomized control trial (RCT), in which the unobserved effects are truly uncorrelated with the variable indicating treatment. RE estimation may be the appropriate choice. Moreover, economists, who more often work with observational data, are often more interested in causal inference, while health practitioners are often more interested in prediction. While it is possible that the disparate choices shown in Figure 1 are derived from fundamentally different data that require different estimators, the research that follows shows that in practice it can be difficult to confidently choose between estimators, especially in small samples. Naively following a discipline-specific norm can lead to biased or inefficient estimates.

In addition to choosing which estimator to use, correctly specifying the variables used for each estimator is important. Traditional RE and FE specifications call for a single outcome variable (*y*) to be regressed on observed explanatory variables (*x*). In many cases, each estimation method transforms the data (discussed below) and ordinary least squares (OLS) is applied to estimate the model. Still, a number of variants of this traditional specification have proven useful. Mundlak argued persuasively in 1978 that, when correctly specified, the RE and FE estimators are effectively the same estimator [29]. Thus, properly specifying a model proves as important as selecting which estimator to use.

With the disparate perspectives on estimation selection and the importance of specification in mind, the objective of this paper is to discuss the theory behind the traditional RE and FE estimators, and to illustrate in detail when each of these estimators is most appropriate via simulation of varied situations. In addition, we consider an alternative specification that uses the RE estimator to achieve estimates asymptotically equivalent to those from FE estimation. This hybrid, called the within-between (WB) approach, is an augmented version of the specification proposed by Mundlak, and retains the best characteristics of traditional RE and FE estimation [7], [30].

We follow Clark and Linzer and use simulation to explain and illustrate the differences between these estimators [31]. Clark and Linzer use simulation to evaluate the Hausman test and compare RE and FE estimation, showing that the Hausman test is neither a “necessary nor sufficient statistic” when choosing between the two traditional estimators. We build upon this work by including a number of additional simulation dimensions, assessing each estimator's ability to predict outcomes (in addition to evaluating each estimator's ability to estimate marginal effects), and evaluating a third estimator – the WB estimator. Due to its infrequent use, the properties of the WB estimator have not previously been explored via simulation. In particular, we shed light on the small sample properties of this estimator, which are not well known. The inclusion of these additional features and a different interpretation of the overlapping results lead to a unique set of recommendations. Furthermore, we frame the discussion in the context of population health as health researchers exploring observational data seem predisposed to using a method that might generate bias.

In the simulation, we create 16,200 unique scenarios, each a combination of important dimensions: the number of groups, the size of the groups, within-group variation, between-group variation, amount of measurement error, amount of autocorrelation, and amount of the variation explained by the covariates. On each simulated dataset, we apply the three estimators and evaluate which estimator has the least biased marginal effect estimates (coefficient estimates) and predictions (fitted outcomes). We apply the Hausman test to each dataset, as well. The Hausman test is the traditional tool used to assist researchers in choosing between the traditional RE and FE estimators.

This paper begins with an explanation of the underlying clustered data model and the traditionally specified RE and FE estimators. We also outline and explain the WB approach. A simulation, described subsequently, illustrates that there is a time and place for each of the three estimators. Unfortunately, the simulation also shows that no single rule can offer infallible guidance when selecting an estimator, although in many cases the WB approach retains the best characteristics of the two traditional estimators. While the Hausman test can be marginally informative, it is only reliable in large samples. Finally, this paper closes with suggestions for when each estimator should be employed.

### Clustered data model

Empirical analyses in health routinely consider groups of clustered individuals, such as households, treatment facilities, and health status groups. It is possible to further cluster these grouped data at higher levels, such as service platforms, states, and countries, or even into disease-endemic regions or income-groups. In longitudinal data, points in time can be clustered together, such that, at a specific time, a shock influences all observations. This would be a second dimension of clustering. Without loss of generality, everything discussed in this paper can be generalized to multiple dimensions of clustering, although exploring this in simulation is beyond the scope of this paper.

While group membership is observed, the actual determinants of the outcome variable are considered unobserved, and in most cases cannot be included in the model. Clustered data become problematic when unobserved group-level characteristics affect the outcome. In these cases, the conditional group-level means of the outcome vary across groups. This characteristic, known as unobserved heterogeneity, violates an assumption necessary for OLS to be the best linear unbiased estimator, leading to inefficient estimation and biased inference (heteroskedastic residuals) and potentially biased estimates [1], [32].

Equation (1) represents an observation from such a model. Here *y* is the outcome variable of interest, *x* is the explanatory variable, *β* is the marginal effect, *ε* is the residual, and *μ* is the single, aggregated, unobserved group-level effect. In reality, it is likely that there are many unobserved group-level effects, but in our case, *μ* can be considered the aggregation of these many effects. (Without loss of generality, a constant should be included, and if appropriate the model can be extended to include many explanatory variables. Here, we abstract to the simplest case.) *ε* is assumed to be independently and identically distributed across the sample. Subscripts *j* and *n*, where 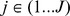 and 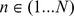, indicate the group and observation identification within each group, respectively. If the data is longitudinal then *n* indicates the time at which each observation is sampled, while *j* indicates the unit observed.
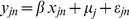
(1)


For estimation, it is possible to ignore the unobserved group-level effect, and consider the unobserved heterogeneity simply as a part of the residual. This is represented in equation (2). Applying OLS to equation (2) is often called the pooled or population average estimator [11]. There are two central concerns regarding the pooled estimator. The first concern is related to bias. Because 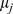 is part of the true data generating process of 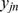 but is ignored in estimation, the pooled estimator can suffer omitted variable bias (OVB). If we rewrite 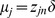 such that *z* is a set of *J-1* binary variables indicating group-membership and 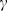 is the vector of effects measuring how group-membership affects 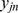, then it can be shown that 
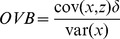
 [11]. Thus, the pooled estimator will be biased unless the included explanatory variables and the (excluded) group-level effects are independent (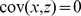). The second common concern related to the pooled estimator is heteroskedasticity. Heteroskedastic residuals are residuals that are not distributed with the same variance across a sample. As a consequence, an estimator suffers from biased inference (the estimated standard errors are too small) and inefficiency (it is less precise that other linear estimators) [1], [11]. These problems occur if the group-level effects varies across the sample (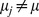 for at least some *j*). In clustered-data it is unlikely that the group-level effect does not vary across groups. Specifically, the OLS pooled estimator will generate standard errors that are too small for between-cluster explanatory variables, and it will generate standard errors that are too large for within-cluster explanatory variables [10].
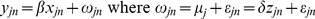
(2)


### Random-effects estimation

If 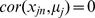 then the pooled estimator is not biased (as OVB  = 0), and the only concern regarding the pooled estimator is the heteroskedastic residuals. An alternative to the pooled estimator that controls for heteroskedasticity is the RE estimator. Like pooled estimation, RE estimation does not explicitly model the unobserved group-level effects, and thus, it is only unbiased if group-level effects are independent from the included explanatory variables [11]. RE estimation assumes 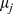 is normally distributed with mean zero and employs feasible generalized least squares (FGLS). FGLS applies OLS to equation (3), and is an efficient method for dealing with heteroskedasticity [12]. In practice, maximum likelihood estimation often replaces FGLS. It is asymptotically equivalent. 
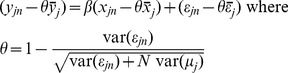
(3)


### Fixed-effects estimation

In some disciplines the term “fixed-effects” is used to mean a marginal effect that is constant across the sample. Using that terminology all the right-hand-side variables from equations (1)–(6) would be considered fixed, because *β* is assumed to be homogenous. Using this alternative terminology, fixed-effects are contrasted with random parameters (or effects), which allow for group-specific marginal effects. For this paper, FE estimation refers exclusively to unobserved group-specific effects that set the groups-specific intercept (the constant). Here, RE estimation refers to estimation with a group-specific intercept.

Unlike the pooled and RE estimators, the FE estimator explicitly models group-level effects. To do this, the FE estimator includes a set of *J-1* binary variables indicating group membership. Each group, save one, has its own indicator. When OLS is applied to equation (4), where *z* is a matrix of *J-1* indicator variables and *δ* is a vector of marginal effects, it is called the least squares dummy variable (LSDV) version of FE estimation [11]. 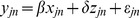
(4)


In practice the LSDV version of FE is often replaced by a numerically equivalent estimator that is less computationally taxing. This estimator regresses *y*, net of the group-specific *y* mean, on *x*, net of the group-specific *x* mean. That is, OLS is applied to equation (5). Equation (5) illustrates why using this transformation accounts for the unobserved group-level effects even though 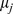, which is unobserved, is not explicitly included. FE estimation differences away all variation between groups and relies completely on variation within groups. This is why the FE estimator is sometimes called the within estimator. The FE estimator fundamentally assesses how changes in *y*, *within* each group, are associated with changes in *x*, *within* each group. 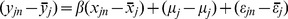
(5)


### Comparing random- and fixed-effects estimation

Both RE and FE estimation rely on the assumptions of OLS. The estimated models (equations (3) and (5), respectively) must be correctly specified, each variable of *x* must be strictly exogenous and linearly independent, and the residual must be independently and identically distributed. When these conditions are met, theory states that FE estimation is unbiased and consistent. RE estimation requires an additional assumption - the group-level effect and the included explanatory variables must be independent in order to avoid OVB. When this assumption is met, RE estimation is unbiased, consistent, and, because it utilized both the within- and between-group variation, efficient. Under this assumption, FE estimation is not efficient because it only utilizes the within-group variation [12]. Thus, the correlation between the explanatory variable(s) and group-level effects distinguishes which of these two estimators to utilize.

However, there has been great confusion on this matter [12], [32]. Determining if group-level effects are random, meaning they are representative of random draws from broader population is of marginal importance, as a (large) number of draws from any cross-section will most likely appear random [12], [29]. More importantly, if 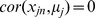 then RE estimation is superior to FE estimation, regardless of whether group-level effects are determined to be random. In these cases, both estimators are unbiased and consistent, but only RE is efficient.

In many observational health studies, even if the sample of interest is randomly drawn from a larger sample, the unobserved effect and included explanatory variables are not independent, 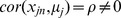. In these cases, the OVB of RE estimation increases with *ρ*. There are many reasons why the unobserved group-level effects and the included explanatory variables might be correlated. When health facilities are clustered into regions, the unobserved group-level effect controls for policies, supply of medicines, disease patterns, and budgets common to each region. It is highly probable that these characteristics that make up the unobserved effect are correlated with variables included in the estimation such as population density or number of physicians. Similarly, in cross-country analyses of population health, it common for researchers to cluster countries into geographic regions to control for unobserved disease patterns and demographic characteristics. It is highly likely that these unobserved group-level characteristics are correlated with even the simplest covariates included in the model such as gross domestic product, baseline population health status, or education levels. In all of these cases, RE estimation is biased.

Unfortunately, knowing RE estimation is biased does not unambiguously guide a researcher to the FE estimator. Even when 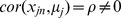, RE estimation remains a more precise estimator than the FE estimator. In most cases, as *ρ* moves away from zero, RE estimation precisely estimates a biased marginal effect. As Rabe-Hesketh and Skrondal show, there are some situations where the RE model can produce the unbiased within-cluster FE estimate. Generally, this occurs when the within-cluster standard error is significantly smaller than the between-cluster standard error, meaning that the RE estimator is weighted toward the within-cluster estimate. This happens with a very large number of observations per cluster, a high *ρ*, or low between-cluster variance in exposure [10]. In these situations, RE is not necessarily more precise or more biased. For this reason, choosing between the two estimators, even if *ρ* is known, is not always simple and revolves around a bias-precision tradeoff. Clark and Linzer state, “The appropriate question to ask… is *how much* bias results – and whether the resulting bias can be justified by the gain in efficiency” [31].

**Figure 2:**
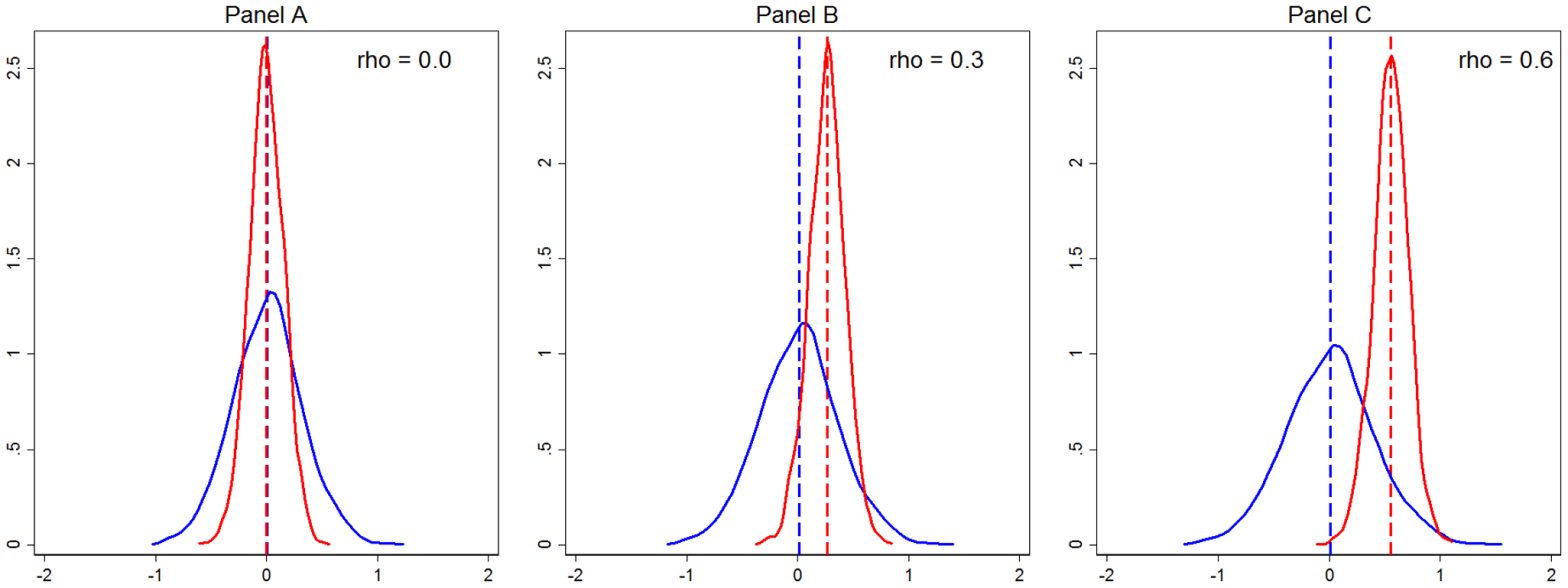
Distribution of errors. Red lines show the distribution of errors from RE estimation, while the blue lines show the distribution of errors from FE estimation. Each panel shows the correlation between the explanatory variable and the group-level effect set to a different value of ρ (0.0, 0.3, 0.6), increasing from left to right. Simulation based on the correct specification of a model with 50 groups and 10 observations per group. 50% of the variation of the outcome variable is explained by residuals, while only 10% of the variation in the explanatory variable is within-groups.

Figure 2 illustrates why assessing the bias-precision tradeoff can be less than straightforward. In each of the three panels, two distributions are shown. For each simulated dataset, the RE estimator (red) and FE estimator (blue) imperfectly estimates the marginal effect, leading to one error 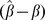 per estimator per simulated dataset. This is repeated 1,000 times on simulated data to create a distribution of 1,000 errors for each estimator. The accompanying dashed vertical lines show the mean of each distribution of errors. Thus, bias is illustrated when the mean error (the dashed vertical line) is not equal to zero. Precision is illustrated by a tight distribution of errors.

Panel (a) of Figure 2 shows cases when 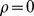, while panel (b) shows cases when 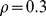, and panel (c) shows cases when 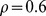. In panel (a), RE is clearly a superior estimator because it is unbiased and more precise (thus, efficient). Conversely, panel (c) shows cases where it is clear that FE is superior because RE is so biased. More ambiguity is present in panel (b). The vertical line right of zero shows that RE is biased upward, as 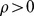, but the variance around the RE errors' mean is much smaller. It is possible that despite the bias, the absolute error from RE estimation is still sometimes smaller than the corresponding FE error. For panel (b), theory is less instructive and the practitioner needs to make a judgment between accepting bias or imprecision.

In addition to weighing the costs of bias and imprecision, there are several other practical differences between the traditional RE and FE estimators. One difference exists because the FE estimator removes all between-group variation and only uses within-group variation. This means that group-level variables (that are constant across the entire group) cannot be included in the estimated model, and, for panel data, time-invariant variables cannot be included. Thus, when the objective of an analysis is to measure the effect of group-level variables, FE estimation is not a viable method. RE estimation, on the other hand, capitalizes on both within- and between-group variation, and therefore allows for the inclusion of variables that are constant within a group.

A second distinction between the two traditional estimators is that RE estimation tends to be more flexible and fits easily into a hierarchical framework. Using this framework, groups are easily nested within one another, variables that affect different levels of a hierarchy are more easily explored, and heterogeneous marginal effects can be explored in a random coefficient context [7], [11]. While greater flexibility is a noteworthy advantage favoring RE estimation, it does not obviate the assumption regarding the independence between the group-level effects and explanatory variable. When this assumption is not met, the traditional RE estimation will be biased.

### The within-between approach

In the traditional specifications just discussed, RE is precise and quite flexible, but is also likely to be biased. Alternatively, FE estimation is unbiased, but less flexible, less precise, and cannot be used to explore the effect of group-level characteristics. In this subsection, we explore one estimation variant meant to marry the two traditional estimators and take advantage of the best characteristics of each. The hybrid we consider has several names, called the “within-between” estimator by some [7], [30], the “Mundlak-approach” by others [11], [33], and the “Mundlak correlated random effects” model by another [34]. This estimator applies RE estimation to equation (6), where 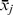 is the group-level mean of each of the included explanatory variables. 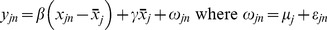
(6)


Mundlak suggested estimating 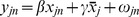 where 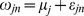, although Bell and Jones (2012) point out three reasons why equation (6) should be preferred. For space, we only consider equation (6) in simulation. When both the cluster-level means, and the deviations from the cluster-level means are included in a model, the coefficient estimate associated with the deviations from the cluster-level means is not correlated with the cluster-level means. Thus the coefficient is not adjusted for the between-cluster effect, and the coefficient reflects both the within and between effect. In order to obtain the correct within-cluster effect from equation (6), the between effect (γ) must be subtracted from the β attached to deviations from the cluster-level means. If the alternative Mundlak specification is used, the correctly adjusted within and between effects are generated.

Some consider the WB approach to be a compromise between RE and FE. While the mechanics applied and versatility are based on RE estimation, the group-demeaning of *x* applies an alternate version of FE estimation. In fact, others have described the WB approach as a hybrid FE model [9]. The WB specification can be estimated using a variety of estimation methods, including ordinary least squares regression with cluster robust standard errors, and generalized estimating equations. The practitioner can execute the WB estimator presented in this paper using standard random effects estimation in any modern statistical or econometric software by using unit-level random effects and specifying the model to include the unit-level means, and deviations from the unit-level means of all explanatory variables.

Group-demeaning ensures that 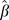 from equation (4), (5), and (6) are asymptotically equivalent [34]. Failure to demean the individual observations will result in biased within effect estimates. 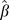 is considered the “within” effect, which assess changes within a group (as the FE estimator does), while 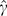 measures the effect of *x* between groups [7]. Most importantly, the explanatory variable and the unaccounted-for group-level effects of equation (6) are fully independent when the group mean is also included as an explanatory variable. Thus, this specification of RE is unbiased, and as Mundlak elegantly concluded, “The whole literature which has been based on an imaginary difference between the two estimators… is based on an incorrect specification [of RE] which ignores the correlation between the [group-level] effects and the explanatory variables” [29].

While the WB approach and FE estimation are equivalent asymptotically, this is not the case in finite samples. Furthermore, using the RE estimator on an augmented specification to derive the standard FE estimates does not solve the bias-precision trade-off. It is still plausible that in some scenarios, a biased RE estimate might be preferred to the unbiased estimate from FE estimation or the WB approach. This possibility is even more likely when samples are small. To explore these concerns, we turn to simulation.

## Methods

To garner insights on the RE and FE estimators and the WB approach, we simulate and draw conclusions from over 16 million datasets. Theory offers limited guidance for adequately addressing the bias-precision tradeoff, and little is known about the small sample properties for the WB approach. While it may sound ideal to never use a biased estimator, Figure 2 (shown above) illustrates that at times using an imprecise estimator might be worse than using a slightly biased estimator.

This section includes an explanation of the dimensions that define each data generating process, an explanation of how each dataset is generated, and an explanation of the metrics used to compare RE, FE, and WB estimators.

### Input dimensions

We consider 16,200 unique combinations of input parameters, simulating 1,000 datasets for each combination. The dimensions adjusted are listed in Table 1. The number of clusters (*J*) ranges from ten to 100 and the number of observations per cluster (*N*) ranges from five to 50. The group-level effect (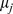) is normally distributed with mean zero and variance one. The single explanatory variable (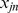) is normally distributed with mean zero and variance (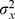). 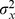 is set to be the same, half, or double the variance of the group-level effect. The variance of the explanatory variable can be disaggregated into the variance that is within groups (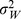) and variance between groups (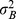), such that 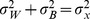. This simulation uses cases where 10% to 90% of 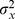 variance is from within groups. The residual (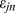) is also normally distributed with mean zero and variance (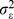), such that 10% to 90% of the variation of *y* is explained by residual. The marginal effect (*β*) of the single explanatory variable is set to one. Most importantly, the correlation (*ρ*) between group-level effect and the explanatory variable is set to range between zero and 0.7. In many cases it is impossible to generate *ρ*>0.7 and maintain the desired values for the within, between, and total explanatory variable variance. In these cases the covariance matrix needed to generate the group-level mean of the explanatory variable that is properly correlated with the group-level effect is not positive semi-definite.

**Table 1:** Dimensions adjusted for simulation.

Input	Baseline value	Adjusted values
Number of groups:	*J* ∈ (10, 50, 100)	*J* ∈ (10, 50, 100)
Number of observations per unit:	*N* ∈ (5, 10, 50)	*N* ∈ (5, 10, 50)
Correlation between fixed effect and explanatory variable:	ρ ∈ (0.00, 0.10,… 0.70)	ρ ∈ (0.00, 0.10,… 0.70)
Correlation between explanatory variable and residual:	ψ = 0	ψ ∈ (0.0, 0.2)
Variation in explanatory variable:	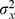 = 1	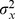 ∈ (0.5, 1.0, 2.0)
Share of the variation in explanatory variable that is within unit:	τ = 0.50	τ ∈ (0.10, 0.25, 0.50, 0.75, 0.90)
Share of the variation in outcome that is due to residual:	π = 0.50	π ∈ (0.10, 0.25, 0.50, 0.75, 0.90)
Coefficient on independent variable:	*β* = 1	*β* = 1
Autocorrelation between residual and previous residual:	υ = 0	υ ∈ (0,0.2)

To include a wide set of plausible scenarios, we also consider two types of model misspecification. The first type of model misspecification sets the correlation between the explanatory variable and residual to be zero or 0.2 (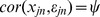). When 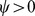 the explanatory variable is endogenous and OLS is biased. In observed data, an endogenous variable can be caused by measurement error, reverse causation (simultaneity), serial correlation, or an omitted variable. The second type of model misspecification induces serial correlation directly into the residual. In these cases, the correlation between residual and the previous within-group residual (assuming longitudinal data) is zero or 0.2.

### Simulating the datasets

To generate the simulated datasets, we follow a four-step process. First, we use *J* and *N* to set the total number of observations, such that *j* and *n* uniquely identify each of the J*N observations. Second, we randomly draw two *J*-length vectors from a multivariate normal distribution, such that:
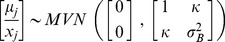
(7)where 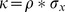, 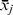 is the group mean explanatory variable, 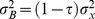, and 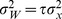. Third, we randomly draw two J*N-length vectors from a multivariate normal distribution such that:
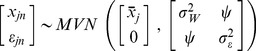
(8)and
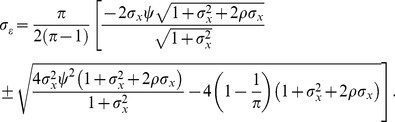
(9)


Equation (9) ensures that the variance of the residual accounts for *π* of the variance of the outcome variable (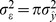). Fourth, the outcome variable 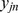 is generated according to equation (1).

### Evaluating RE and FE estimation

For all 16,200 scenarios, we apply three estimators: the traditional RE estimator, the traditional FE estimator, and the WB estimator. Then, for each estimator, we measure the error of the estimated marginal effect 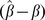. We calculate the distributions of the 1,000 errors (mean, 2.5^th^ percentile, and 97.5^th^ percentile) and the mean squared error (MSE). MSE is a valuable summary statistic because it penalizes an estimate for both bias and inefficiency, such that 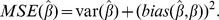


We also evaluate how well each estimator predicts values of the outcome variable (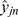). For each simulation, we calculate the root mean squared error (RMSE) of the predicted values for each simulation. For each estimator and combination of dimensions, we calculate the distribution of the 1,000 RMSE (mean, 2.5^th^ percentile, and 97.5^th^ percentile).

Finally, for each simulation we also conduct the Hausman test [35]. The Hausman test is the conventional tool used to guide practitioners towards or away from the RE estimator. The test is based on the intuition that if the estimated marginal effects of RE and FE are not statistically different then both estimators must be unbiased and consistent. This conclusion is drawn from the fact that FE is unbiased and consistent; if the estimated marginal effects are not statistically different it is reasonable that the omitted variable bias that could corrupt RE is negligible. Thus, the null hypothesis of the Hausman test is that both RE and FE are consistent. If the null hypothesis cannot be rejected, then conventional wisdom suggests RE estimation should be applied because it is efficient. However, if the null hypothesis is rejected, conventional wisdom suggests FE estimation. Stata 12.0 was used for all simulations and analyses, and code is available in File S1.

## Results

### Baseline results

**Figure 3:**
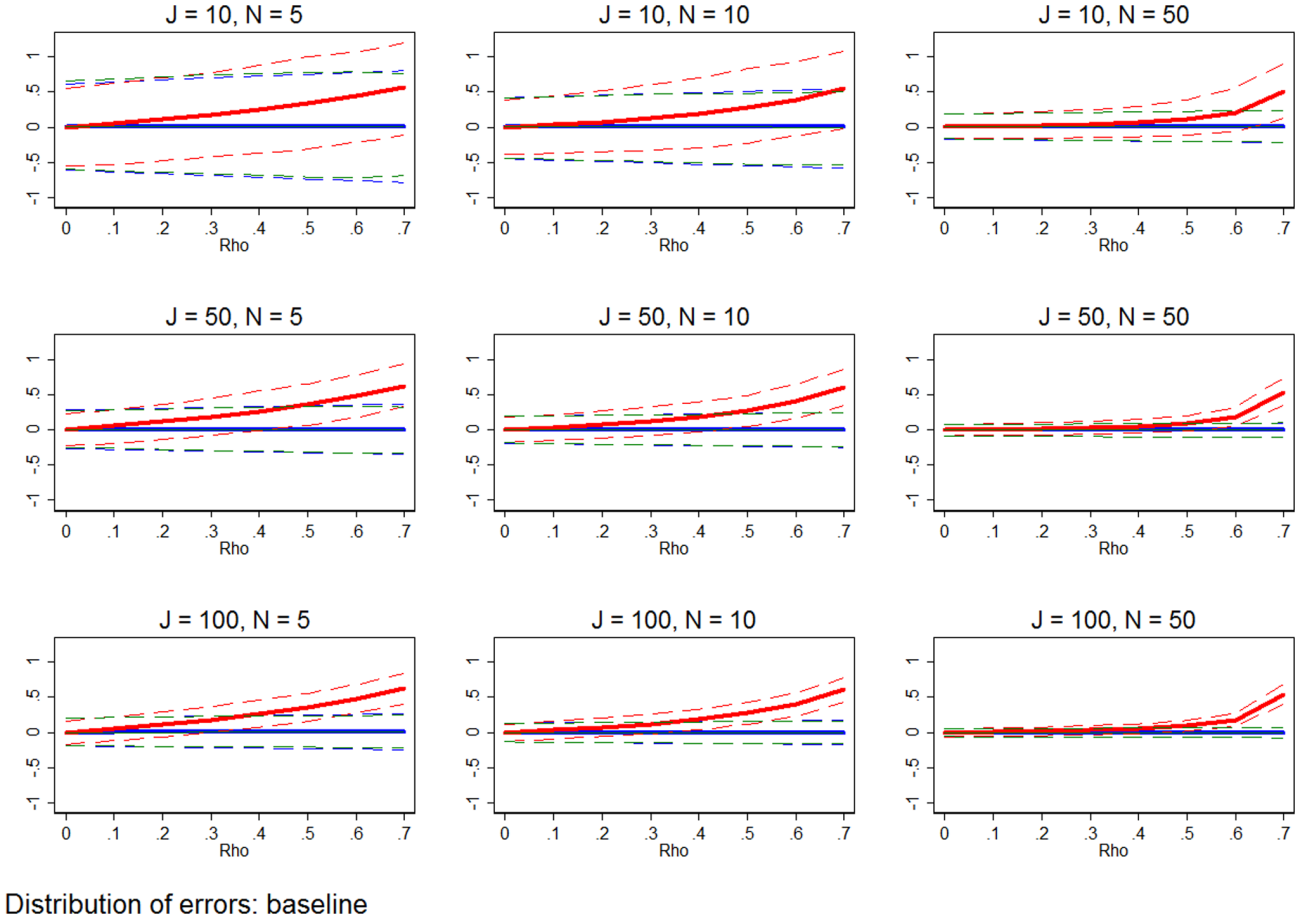
Distribution of errors of estimated marginal effects at baseline specification. The solid red line shows the mean error in the marginal effect estimates from RE estimation, while the dashed red lines show the 95% range of the RE estimation errors. The solid blue line and dashed blue lines show that mean and 95% range of the errors from FE estimation. The solid green line and dashed green lines show that mean and 95% range of the errors from the WB approach. All simulation inputs are baseline.

We start by comparing the RE, FE, and WB estimators using our “baseline” simulation setup, altering only the correlation between the group-level effects and the included independent variables (*ρ*), the number of clusters (*J*), and the number of observations per group (*N*). The baseline inputs are listed in Table 1. At the baseline, the group-level effect's variance (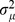) and explanatory variable's variance (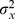) are set to one. We disaggregated 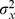 evenly, such that the between-group variance (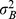) and within-group variances (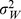) are each set to 0.5 (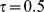). The baseline setup also assumes that the model is correctly specified (no endogeneity or serial correlation is induced, 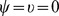) and 50% of the variance of the outcome variable can be explained by the group-level and explanatory variable (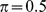). For comparability, Figures 3 through 11 of this paper modeled after Clark and Linzer's original graphs [31]. These graphs are an effective and efficient method to displace a host of results.

**Figure 4:**
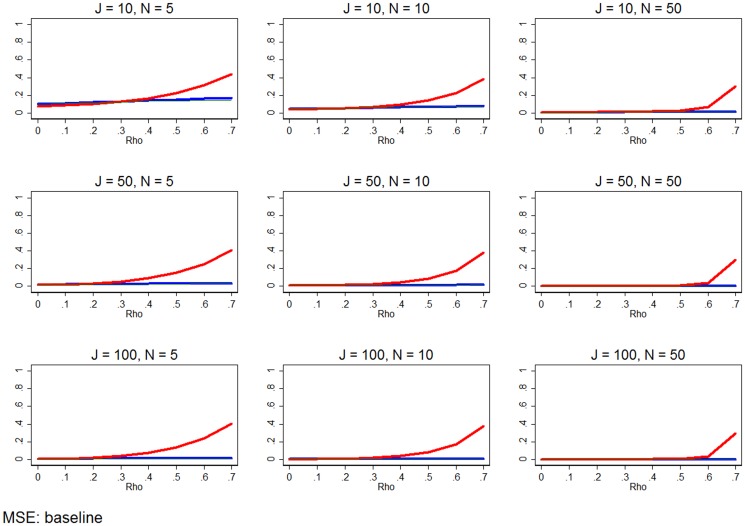
MSE of marginal effect estimates at baseline. The red line shows the MSE from the errors in the marginal effect estimates from RE estimation. The blue and green lines shows the same for FE estimation and WB approach, respectively. All simulation inputs are baseline.

**Figure 5:**
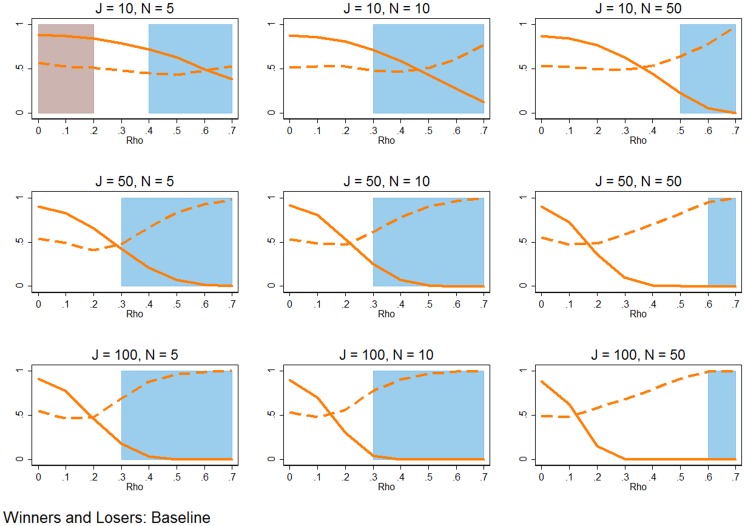
Hausman test. The solid orange lines show the share of the simulations for which the Hausman test does not reject, at the 90% confidence level, the null hypothesis that both RE and FE estimation are consistent. Conventional wisdom is that this suggests that researchers should use RE estimation as it is more efficient. The dashed orange lines show the share of the simulations for which the Hausman test suggests the estimator with smaller absolute error. The red background indicates when the RE estimator is MSE-preferred, while the blue background indicates when the FE estimator is MSE-preferred. The white regions indicate that the difference between the MSE of the two estimators is trivial. All simulation inputs are baseline.

**Figure 6:**
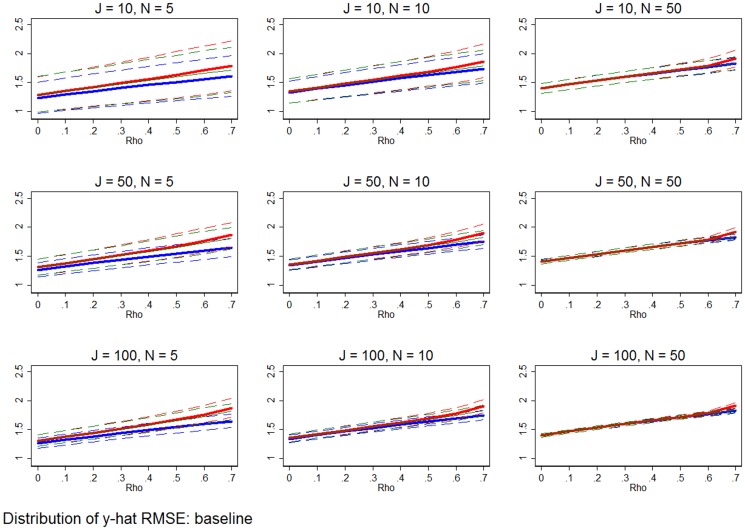
Distribution of RMSE from predicted outcomes. The red lines show the mean and 95% confidence interval of the RMSE derived from the fitted values using RE estimation. Each combination of inputs is made up of 1,000 simulations, and each receives its own RMSE based on the errors of the fitted values. The blue lines show the mean and 95% range of the RMSE acquired using the FE estimator. The green lines show the mean and 95% range of the RMSE from the WB approach. All simulation inputs are baseline.

**Figure 7:**
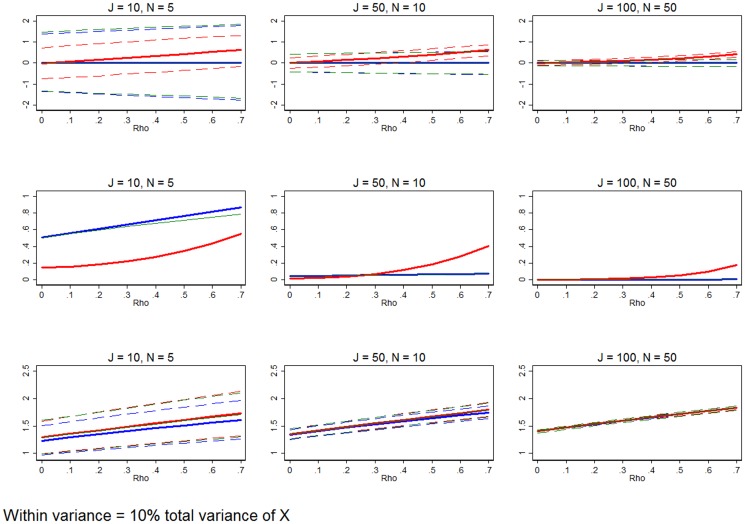
Significant between-group variation relative to within-group variation. Row 1 (interpreted like Figure 3) shows the distribution of the errors in marginal effects estimates from the RE estimation (red), FE estimation (blue), and WB approach (green). Row 2 (interpreted like Figure 4) shows MSE associated with the RE estimation (red), FE estimation (blue), and WB approach (green) errors. Row 3 (interpreted like Figure 6) shows the distribution of the RMSE from the fitted values estimated using RE estimation (red), FE estimation (blue), and WB approach (green). The between-group variation is set to 0.9, while the within-group variation is 0.1. All other simulation input parameters are set to baseline.

**Figure 8:**
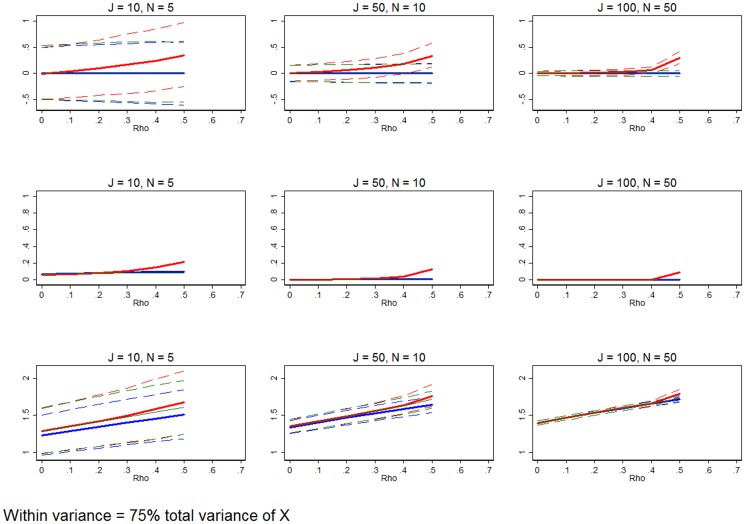
Significant within-group variation relative to between-group variation. Row 1 (interpreted like Figure 3) shows the distribution of the errors in marginal effects estimates from the RE estimation (red), FE estimation (blue), and WB approach (green). Row 2 (interpreted like Figure 4) shows MSE associated with the RE estimation (red), FE estimation (blue), and WB approach (green) errors. Row 3 (interpreted like Figure 6) shows the distribution of the RMSE from the fitted values estimated using RE estimation (red), FE estimation (blue), and WB approach (green). The within-group variation is set to 0.75, while the between-group variation is 0.25. All other simulation input parameters are set to baseline.

**Figure 9:**
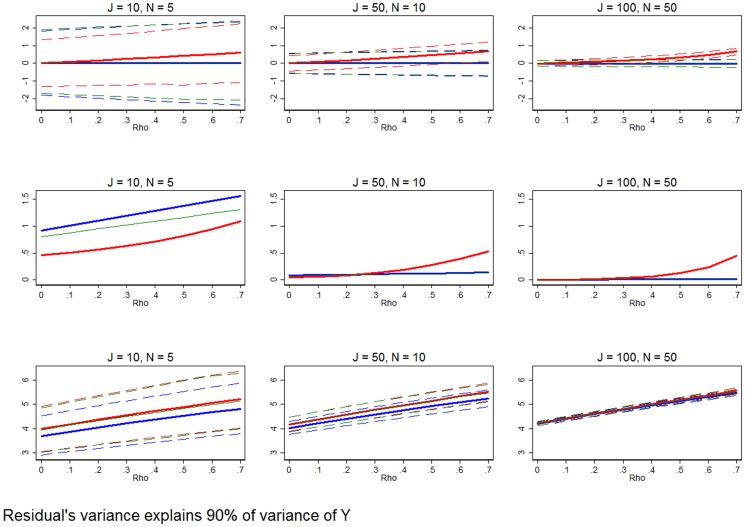
Poorly fit model that explains only a small portion of the outcome variable's variance. Row 1 (interpreted like Figure 3) shows the distribution of the errors in marginal effects estimates from the RE estimation (red), FE estimation (blue), and WB approach (green). Row 2 (interpreted like Figure 4) shows MSE associated with the RE estimation (red), FE estimation (blue), and WB approach (green) errors. Row 3 (interpreted like Figure 6) shows the distribution of the RMSE from the fitted values estimated using RE estimation (red), FE estimation (blue), and WB approach (green). The variance of the residual is set such that it explains 90% of the variation of the outcome variable. All other simulation input parameters are set to baseline.

**Figure 10:**
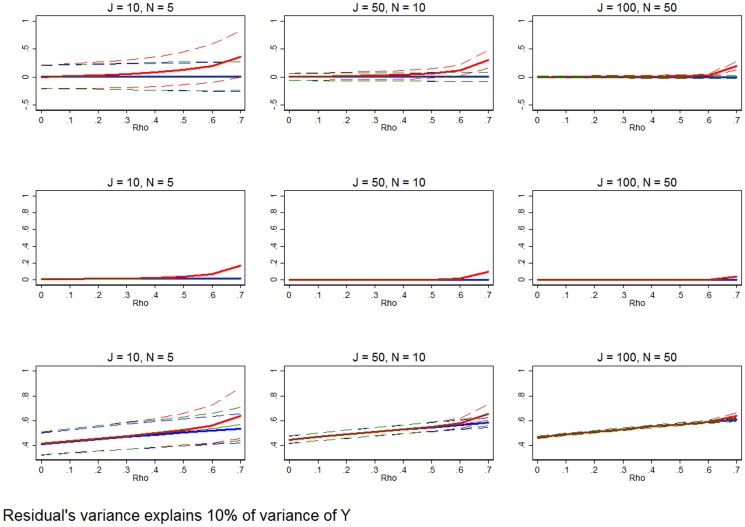
Well fit model that explains a significant portion of the outcome variable's variance. Row 1 (interpreted like Figure 3) shows the distribution of the errors in marginal effects estimates from the RE estimation (red), FE estimation (blue), and WB approach (green). Row 2 (interpreted like Figure 4) shows MSE associated with the RE estimation (red), FE estimation (blue), and WB approach (green) errors. Row 3 (interpreted like Figure 6) shows the distribution of the RMSE from the fitted values estimated using RE estimation (red), FE estimation (blue), and WB approach (green). The variance of the residual is set such that it explains only 10% of the variation of the outcome variable. All other simulation input parameters are set to baseline.

**Figure 11:**
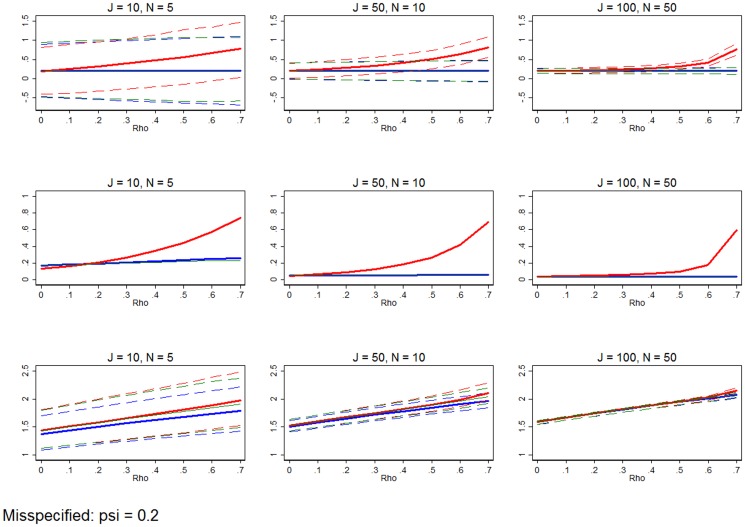
Misspecified model. Row 1 (interpreted like Figure 3) shows the distribution of the errors in marginal effects estimates from the RE estimation (red), FE estimation (blue), and WB approach (green). Row 2 (interpreted like Figure 4) shows MSE associated with the RE estimation (red), FE estimation (blue), and WB approach (green) errors. Row 3 (interpreted like Figure 6) shows the distribution of the RMSE from the fitted values estimated using RE estimation (red), FE estimation (blue), and WB approach (green). The correlation between the explanatory variable and the residual is set to 0.2. All other simulation input parameters are set to baseline.

Figure 3 assesses the distribution of errors for the baseline setup. In Figure 3, and all the subsequent figures, the x-axis measures *ρ*, red is used for the RE estimation, blue is used for FE estimation, and green is used for WB approach. The y-axis shows the error of the marginal effect estimate 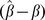. The solid line is the mean of the 1,000 errors, while the space between the dashed lines shows the 95% spread of the errors resulting from each estimator. Each of the nine graphs has a different number of groups (*J*) and observations per groups (*N*). In Figure 3, bias exists when the mean of the distribution of errors is not zero. The distribution of the errors, that is, the width (up and down) of the band surrounding the mean error, serves as a measure of precision.

Three important insights are confirmed in Figure 3. First, the solid blue and green lines are always at zero, meaning the FE estimator and WB approach are always unbiased. Second, RE estimation is more precise than the two competing estimators. The variance of errors around the mean is smaller for the RE estimator than for either of the other approaches. Third, as *ρ* increases, RE estimation becomes biased. The direction of the bias is the same as the direction of the correlation between the group-level effects and the explanatory variable, and symmetric set of graphs could be made with *ρ*<0, resulting in downward bias of the RE estimator. These points are confirmed at all sample sizes, but are clearest and most extreme in the smallest samples.

Figure 3 suggests that it might be advantageous for a practitioner to use the more precise RE estimator in some instances, even if it is biased. For example, the *J* = 10, *N* = 5 and *ρ* = 0.1 case illustrates some scenarios where the bias might be small enough that the practitioner would prefer accepting the bias rather than deferring to the less precise FE estimator.

Figure 4 makes this point more clear. This figure shows the MSE from the 1,000 simulations for each estimator and *J*, *N*, and *ρ* combination. Figure 4 shows results using the same baseline simulation inputs, varying *J*, *N*, and *ρ*. The MSE is a valuable metric because it combines bias and precision into a single metric, and thus can be used as a guide for dealing with the bias-precision tradeoff. In Figure 4, when the red line is greater than the blue and green lines, RE estimation is MSE-preferred, even if it is slightly biased. Based on this metric, Figure 4 shows that in most baseline cases there is either negligible difference between RE, FE, and WB approaches or FE and the WB approaches are clearly MSE-preferred.

Figure 5 illustrates whether the Hausman test is a valuable guide for determining which estimator to select when *ρ* is unknown (as it is in most cases). The backgrounds of the graphs in Figure 5 are based on the MSE estimates graphed in Figure 4. The background of Figure 5 is red if RE is MSE-preferred. If FE estimation is MSE-preferred, then the background of Figure 5 is blue. If the MSE of the two estimators are within 0.005 of each other, we consider this a trivial difference and leave the background white.

In Figure 5, the solid orange lines shows the share of the 1,000 simulations for which the Hausman test did not reject the null hypothesis, using α = 0.1. In other words, the solid orange line shows the share of the 1,000 simulations that the Hausman test recommended the standard specification of RE estimation. If the Hausman test is an effective test, the orange line will be near 1 (100%) when the background is red, and near zero when the background is blue. The dashed orange lines of Figure 5 show the share of the 1,000 simulations where the Hausman test recommended estimator with the smallest absolute error (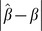), comparing only the traditional specifications of RE and FE. This metric assumes that the practitioner prefers to minimize absolute error and has a binary choice between traditional RE and FE estimation. When the dashed orange line is near one it indicates that the Hausman test recommended the “better” estimator nearly 100% of the time.

Figure 5 confirms that the Hausman test is most effective in large samples. In small samples, the test frequently fails to reject the null hypothesis even at relatively large *ρ*. As a result, the Hausman test suggests the “better” estimator roughly 50% of the time. As the sample size increases, especially in *J*, the test is more effective. In these cases, the Hausman test is more apt to reject the null as *ρ* increases and the frequency with which the test recommends the “better” estimator moves towards 100%.

Figure 6 shows that FE estimation is better at predicting outcomes. The basic setup of Figure 6 is the same as the earlier figures, though in this case the y-axis measures the distribution of 1,000 RMSE, where a RMSE statistic is derived for each estimator and each simulation based on the error of the J*N predicted values (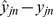). The solid line shows the mean of the 1,000 RMSE, while the pairs of dashed lines show the 95% range of RMSE, for each estimator. No matter what the combination of *J*, *N*, and *ρ*, FE estimation is a better predictor. The mean RMSE and variance of the RMSE are both smallest for FE estimation (relative to RE estimation and the WB approach), meaning that the FE estimator is a less biased and efficient predictor.

### Varying between-group and within-group variance

Figure 7 evaluates the effect of adjusting the explanatory variable's variance such that the majority of 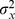 is caused by between-group variation. This adjustment leaves only a small portion of 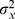 within groups. For Figure 7, we leave 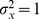 but increase 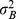 to 0.9 and decrease 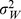 to 0.1 (by setting 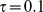). This is characteristic of data with a great deal of (observed) heterogeneity between groups and little change within groups. Examples of such data could include antenatal care within a region, health facilities' expenditure over time, or countries' rate of maternal mortality over time. If the data is longitudinal, this could be considered “sluggish” data [31].

Considering three (instead of nine) combinations of *J* and *N*, Figure 7 illustrates the distribution of the errors of the marginal effect estimates (row 1), the MSE of the estimated marginal effects (row 2), and effectiveness of each estimator at predicting the outcome variable (row 3). Row 1 shows that with small sample size, RE estimation is much more precise than the FE estimation and WB approach. This is because RE is using both between- and within- group variation, whereas FE uses only within-group variation and the coefficient being evaluated in the WB approach measures only the within-group effect. At the smallest sample size, row 2 shows that RE is always MSE-preferred, despite being biased when *ρ*>0. However, the largest sample size (*J* = 100, *N* = 50) shows that the differences between RE, FE, and WB estimation are either negligible or FE estimation and the WB approach are MSE-preferred. Between these two extremes, the medium sized sample (*J* = 50, *N* = 10) exhibits more ambiguity. Row 3 confirms that FE remains marginally, but unambiguously, a better predictor of outcome variables regardless of *J*, *N*, and *ρ*.

While not shown here, the Hausman test offers only marginal insight for these cases, especially when the sample size is small to moderate. This has been recently documented elsewhere in this literature [36]. In these simulations, the test recommends the “better” estimator 55%, 54%, and 74% of the time, for the three sample sizes illustrated in Figure 7. At small to moderate sample size scenarios (with observations less than or equal to 500), the Hausman test offers limited guidance, except when *ρ* is exceptionally large. As the sample size increases past 1,000 observations, the Hausman test can be relied on more heavily.

Figure 8 illustrates the baseline setup adjusted so that 75% of the variation of the explanatory variable is found within groups, while only 25% of the variation is between groups. This type of data is characteristic of health outcomes grouped by facility, national mortality rates grouped by region, or HIV prevalence over time. In these settings there is a great deal of heterogeneity within the group. Figure 8 shows that except for the smallest samples with *ρ* at or very near 0, FE estimation and the WB approach preform as well as or better than RE estimation.

### Varying the amount of variance explained by the model

Figures 9 and 10 vary the model fit. That is, we now consider results where we vary the share of the variance of the outcome variable that is explained by model (*π*). Returning all other inputs back to the baseline level, we set *π* = 0.9, which sets the variance of the residual so that the estimated model is equipped to measure only 10% of the variation of *y*. This data would be characteristic of any model that is poorly fit, with a relatively small R^2^ (coefficient of variation). Figure 9 shows that in small samples, RE estimation clearly outperforms FE estimation and the WB approach, and the RE estimator is unambiguously MSE-preferred, regardless of *ρ*. As the sample size grows, especially in *J*, FE and the WB approach become more plausible estimators. In the largest sample it is clear that FE and the WB approach are either equivalent to RE estimation or MSE-preferred.

Figure 10 considers a model that is exceptionally well fit with the residual only explaining 10% of the variance of *y*. In these cases, FE and the WB approach are clearly equivalent or MSE-preferred, relative to RE estimation, even in the smallest samples. Row 3 shows that while FE remains a better predictor of outcome variables, it is only at the smallest margin.

### Varying all the other dimensions

Figure 11 examines simulations with the baseline setup, but *ψ* is adjusted to 0.2 so that the explanatory variable is endogenous. This is characteristic of data suffering from measurement error, reverse causation, serial correlation, or an omitted explanatory variable that is correlated with an included explanatory variable. What is unique about Figure 11 is that we see for the first time that the FE estimator and WB approach are biased, as the necessary assumptions of OLS are not met. Still, the endogeneity of *x* does not change our primary baseline findings, except for very small samples, where FE estimation and the WB approach are effectively equivalent or MSE-preferred relative to RE estimation. Simulation shows that varying the variance of the explanatory variable or inducing serial correlation into the residual has trivial effects on the choice between the three estimators. Across all these simulations, the baseline results hold.

### Comparing traditional fixed effects estimation and the WB approach

Asymptotically the FE estimator and WB approach are equivalent, as both are consistent. But, the two estimators are not necessarily equivalent in finite samples. Figures 5 through 11 show that FE estimation is an unambiguously superior predictor. The mean RMSE for each scenario is smaller for the FE estimator than for the WB approach. Furthermore, the distribution of RMSE is tighter for the FE estimator, meaning it is also a more precise predictor.

The same cannot be said regarding the estimated (within-group) marginal effects (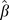). Figures 3 and 6 through 11 show that the distribution of the errors appear to be identical, but comparing the MSE suggests that in small samples the two estimators are not the same. To quantify this difference, we compare the FE estimation's MSE to that of the WB approach for each of the 16,200 scenarios. As the number of observations increases, the estimates from the two estimators should converge towards each other and towards the true marginal effect. In small samples, we see that the difference between the two MSE is not zero, nor centered at zero. For 40% of the simulations with less than 500 observations, the MSE of the FE estimator is greater than that of the WB approach by more than 0.01. Moreover, the MSE of the WB approach is greater than that of the FE estimator by more than 0.01 in 0% of these simulations. This means that in a disproportionally large amount of small sample scenarios, the WB approach garners a smaller MSE.

To explore this further, we look at the pairwise correlation between the simulation input parameters and the difference between the WB approach's MSE and the FE estimator's MSE, assessing only scenarios with less than 500 observations. Table 2 shows that several input parameters are correlated with the difference in MSE, statistically significant at the 99% confidence level. The most important predictor of a smaller WB approach MSE is the number of groups and number of observations per group; as either increases, the difference between the WB MSE and FE MSE moves towards zero; that is, the efficiency gains of the WB method are negligible in large samples. The next two determinants most correlated with the difference in MSE are the share of variation within versus between groups and the goodness-of-fit of the model. As the share of the variation in the explanatory variable shifts to be more within-groups, the difference between the two estimators moves towards zero. Additionally, as the model fit worsens, the two estimators become more equal. To summarize these points: the FE estimator and WB approach are generally very similar, though in small samples the WB approach is generally superior at estimating within-group marginal effects. This superiority is especially true the smaller the sample size, the larger the share of the variation between groups, and the better the model fit.

**Table 2:** Correlation between WB MSE minus FE MSE and simulation input parameters.

	*J*	*N*	*ρ*	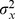	*τ*	ψ	*π*
Correlation	0.1716*	0.1682*	−0.0661*	0.034	0.1738*	−0.0613*	−0.2554*
p-value	0.0000	0.0000	0.0000	0.0128	0.0000	0.0000	0.0000

Pairwise correlation. Asterisk suggests the correlation is significant at the 99% confidence level.

## Discussion

Theory offers one significant piece of guidance: the bias of RE is directly related to  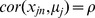. Thus, when 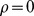, RE estimation is the obvious choice. In most observational studies, however, it seems likely that 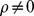. In most cases, it is not difficult to imagine why some correlation between the group-level effects and the explanatory variables will bias the marginal effect estimation. When it is impossible to conclude with confidence that 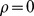, choosing between the FE and RE estimators proves challenging. Researchers are forced to choose between a potentially *precise biased* estimator and an *imprecise unbiased* estimator. Little convention exists about how to balance this bias-precision tradeoff and properly specify estimation. Fortunately, simulation can offer some guidance. We derived five simple and practical rules-of-thumb from this set of simulations.

First, if the purpose of an analysis is prediction (as opposed to inference on marginal effects), then FE estimator is the unambiguously preferred estimator. Figure 6 and row 3 of Figures 7, 8, 9, 10, 11 show that the FE predictions always have a smaller RMSE. Because the FE estimator cannot predict outcomes for groups that are not included in the original estimation, the WB approach is the clear second-best estimator in these situations.

Second, when the purpose of an analysis is inference on marginal effects, simulation suggests the WB approach is preferred over traditional FE estimation. The simulation confirms that the within-group marginal effects estimates are asymptotically identical, but the WB approach provides researchers with additional information regarding the between-group marginal effects (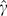). For the small samples included in our analyses, the WB approach consistently has (essentially) equivalent or smaller MSE than the FE estimation. Outside of the variation that can be attributed to noise, there are no scenarios when FE is MSE-preferred over the WB approach.

Third, as a general rule, the larger the sample size, the more a practitioner should avoid traditional RE estimation. Applying FE estimation on all simulated samples with greater than 500 observations led to a median absolute error of 4% of the true marginal effect. RE estimation led to a median absolute error of 8% of the true marginal effect. In simulations with more than 1,000 observations, RE estimation was only MSE-preferred beyond a trivial threshold (0.005) in a very few cases where 90% of variation of *y* could not be explained by the model (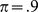). It is important to note that this rule contradicts researchers' tendency to use RE estimation for problems with large *J*. Presumably researchers are hesitant to “waste” degrees-of-freedom for each of the *J* groups when *J* is quite large. While it is true that more degrees of freedom are used by choosing FE estimation, simulation shows that using that FE estimation is MSE-preferred (relative to RE estimation) in most large-*J* scenarios. This is a case where the WB approach is a valuable compromise between the two traditional estimators. The WB approach generates practically equivalent estimates as FE but uses only a fraction of the degrees-of-freedom.

Fourth, small samples mark the circumstances under which a practitioner might consistently choose precision over bias. Our simulations show that, when combined with small samples (of less than or equal to several hundred observations), two observable data characteristics make it especially likely that RE estimation would be MSE-preferred. One scenario is when the estimated model explains a very small portion of the variation in the outcome measurement. When small sample size is combined with a poorly-fit model, the imprecision of FE and WB estimation tends to mislead the researcher more than the bias of RE estimation, even at large *ρ*. The goodness-of-fit of the clustered model can be explored by examining the R^2^ statistic associated with LSDV estimation. Considering only simulations with R^2^<0.5 and less than 500 observations, the traditional RE estimator had a smaller absolute error than the FE estimator 57% of the time.

Another unique scenario when the RE estimator is consistently MSE-preferred and should be considered is for small samples that have relatively small within-group variation for the variable of interest. Again, in these cases, the imprecision of the FE and WB estimators might be more caustic than the RE estimator's bias. In simulation cases with less than 500 observations and within-group variation less than 20% of the total variation, RE estimation leads to a smaller absolute error 53% of the time. However, with small samples, both the WB and FE estimators are less reliable as they draw inference from too few observations and likely too little variation. If choosing a RE model, there may be a large but imprecise distinction compared to FE and WB. The practitioner may have inadequate power to adequately adjust for cluster-level confounding.

The fifth rule garnered from this simulation is that the Hausman test is most insightful with large samples. Unfortunately, these are the same cases when the test is needed the least. In simulations with greater than or equal to 1,000 observations, the Hausman test recommends the estimator with the smaller error 67% of the time. However, when the FE estimator is blindly applied to all these same cases, it generates the same median absolute error and is the “better” estimator (when compared to the traditional RE estimator) in 70% of the simulations.

These rules of thumb do not apply to all situations that the practitioner may encounter. For example, different estimation techniques are required for binary response models and complex survey data. When the outcome variable is binary, specifying RE, FE, or WB model in this framework produces new challenges for correct model specification and interpretation of marginal effects. The RE binary outcome model is a special form of the population average model. Instead of estimating 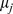 (as in the linear case), it assumes u_j_ has a parametric distribution and is independent from 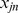 [9]. The marginal effect from a RE binary response model is the population average effect for an individual at 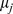 = 0 [12].

The problem of eliminating 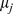 for a FE binary response model is not solved by adding individual indicators, as this would generate the incidental parameters problem [9]. Conditional likelihood methods exist to eliminate 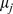 in the logit model, but not in the case of the probit or complementary log – log model [9], [37]. The WB approach can also be implemented by including unit-specific means and the deviations from unit-specific means of each explanatory variable in a RE framework [17]. If using the WB approach with a binary outcome, other link functions, such as probit and complementary log – log, can be used [9].

Also not discussed here is the issue of complex survey data. Since complex sampling designs incorporate unequal selection probabilities, the standard FE, RE, and WB estimators may lead to biased estimates. To remedy this problem, design weights must be incorporated into the likelihood function [38]. We direct the practitioner to the many studies concerning this issue for further discussion of theory and practice [38]–[43].

In many disciplines, the distinction between RE and FE estimation receives little attention. When discussed, attention seems to focus on the relatively irrelevant distributional assumptions or the Hausman test, and too often neglects the bias-precision trade-off. When the RE estimator is specified using the WB approach, a researcher achieves unbiased estimates asymptotically equivalent to FE estimation. Furthermore, since the WB approach operates using RE estimation, it has a flexible environment, with options extending to nested groups, hierarchical models, and random coefficients. Moreover, there is some evidence that the WB approach is also appropriate for non-linear estimation [34]. Understanding these three estimators and their specifications and when to use each is of the utmost importance in conducting rigorous, precise, and unbiased health analyses.

## Supporting Information

File S1 **Stata code to replicate simulation results.**
(PDF)
