# Correction: Random-Effects, Fixed-Effects and the within-between Specification for Clustered Data in Observational Health Studies: A Simulation Study

**DOI:** 10.1371/journal.pone.0156508

**Published:** 2016-05-24

**Authors:** Joseph L. Dieleman, Tara Templin

The authors were notified of a coding error that introduced several errors in the article. The original simulation used the hypothesized group mean instead of the “observed” group mean after randomly drawing from the distribution. This caused the fixed effects estimator to outperform the within-between estimator in small samples. S1 File contains the coding error. Please view the correct S1 File below.

The authors would like to address the “Comparing traditional fixed effects estimation and the WB approach” subsection within in the Results section. The two estimators are equivalent in finite samples. The estimated within effects are identical after the coding mistake is remedied. This means that the mean RMSE and the distribution of the RMSE is identical. The estimated within-group marginal effects are identical. The MSE suggests that the in small samples the estimators are the same. The WB approach does not garner a smaller MSE. Evaluating the pairwise correlation between input parameters and the difference in MSE is no longer relevant. Thus this subsection and Table 2 are no longer relevant.

There are also errors in the text. In the second sentence of the “Results” section of the Abstract should be: In finite samples, the WB and FE estimators are equivalent.

The first sentence of the final paragraph of the Introduction should be: In finite samples, the WB and FE estimators are equivalent.

The second to last sentence of the third paragraph of the Discussion section should be: The two estimators are equivalent in finite samples.

Additionally, there are errors in Figs 7, 9 and 11. Please see the corrected figures here.

**Fig 7:**
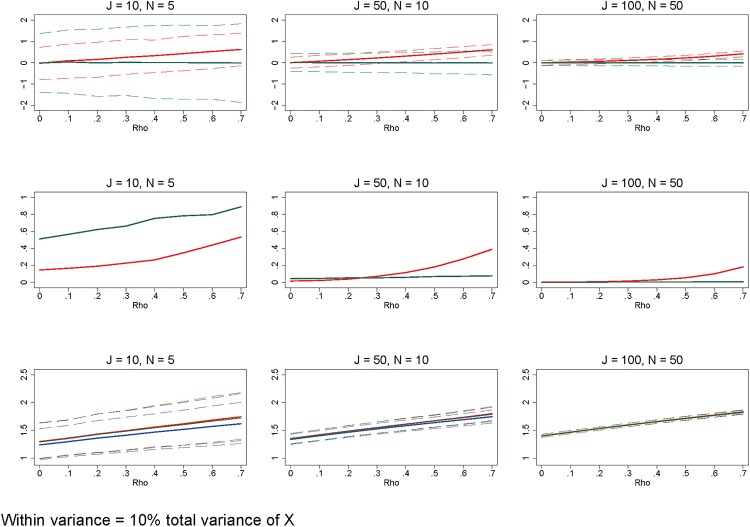
Significant between-group variation relative to within-group variation. Row 1 (interpreted like Fig 3) shows the distribution of the errors in marginal effects estimates from the RE estimation (red), FE estimation (blue), and WB approach (green). Row 2 (interpreted like Fig 4) shows MSE associated with the RE estimation (red), FE estimation (blue), and WB approach (green) errors. Row 3 (interpreted like Fig 6) shows the distribution of the RMSE from the fitted values estimated using RE estimation (red), FE estimation (blue), and WB approach (green). The between-group variation is set to 0.9, while the within-group variation is 0.1. All other simulation input parameters are set to baseline.

**Fig 9:**
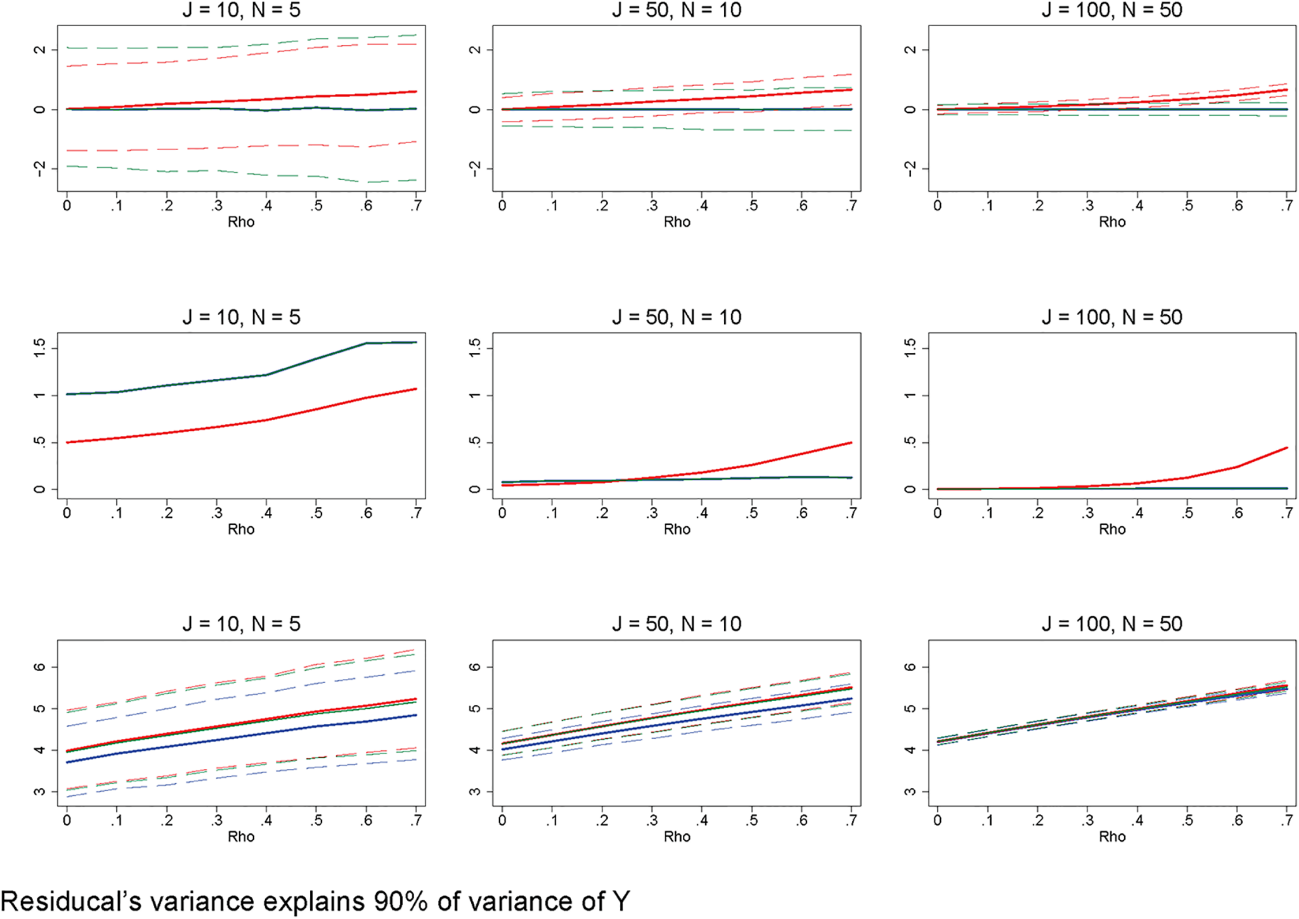
Poorly fit model that explains only a small portion of the outcome variable's variance. Row 1 (interpreted like Fig 3) shows the distribution of the errors in marginal effects estimates from the RE estimation (red), FE estimation (blue), and WB approach (green). Row 2 (interpreted like Fig 4) shows MSE associated with the RE estimation (red), FE estimation (blue), and WB approach (green) errors. Row 3 (interpreted like Fig 6) shows the distribution of the RMSE from the fitted values estimated using RE estimation (red), FE estimation (blue), and WB approach (green). The variance of the residual is set such that it explains 90% of the variation of the outcome variable. All other simulation input parameters are set to baseline.

**Fig 11:**
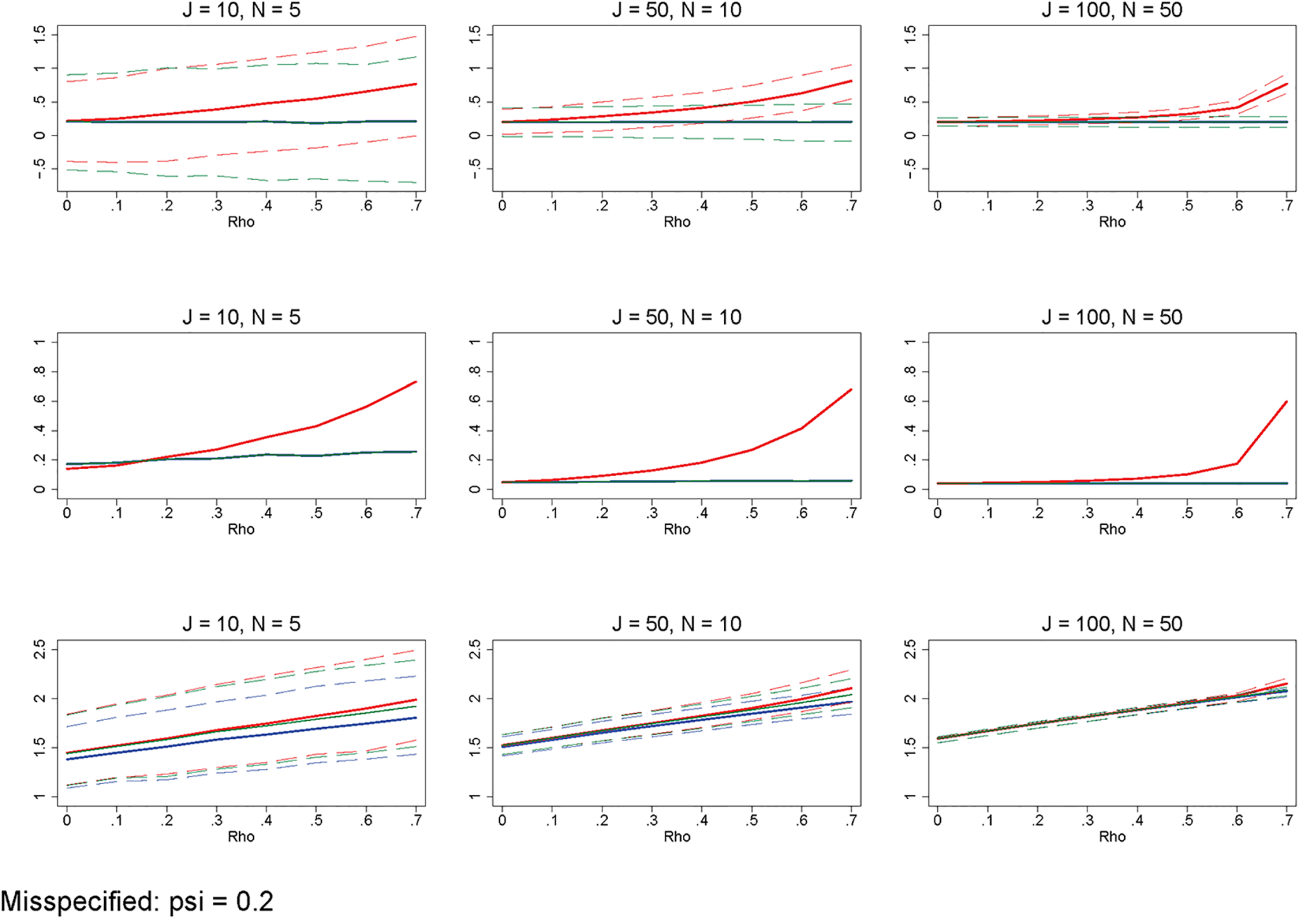
Misspecified model. Row 1 (interpreted like Fig 3) shows the distribution of the errors in marginal effects estimates from the RE estimation (red), FE estimation (blue), and WB approach (green). Row 2 (interpreted like Fig 4) shows MSE associated with the RE estimation (red), FE estimation (blue), and WB approach (green) errors. Row 3 (interpreted like Fig 6) shows the distribution of the RMSE from the fitted values estimated using RE estimation (red), FE estimation (blue), and WB approach (green). The correlation between the explanatory variable and the residual is set to 0.2. All other simulation input parameters are set to baseline.

## Supporting Information

S1 File Stata code to replicate simulation results. (PDF)
