# Supplementary material for: Random-Effects, Fixed-Effects and the within-between Specification for Clustered Data in Observational Health Studies: A Simulation Study
Source: PLoS One. 2014 Oct 24;9(10):e110257. doi: 10.1371/journal.pone.0110257 (PMC4208783; doi:10.1371/journal.pone.0110257)
Supplement: File S1 — Stata code to replicate simulation results. (PDF) [file pone.0110257.s001.pdf]

```
** ************************************************************************************************************************
** Author: Joe Dieleman and Tara Templin
** File Created: March 2013 and updated in August 2014
** Objective: Simulate one dataset for each combination of features, apply RE,FE, WB estimation and the Hausman test to each dataset, and save results in a postfile.
** Structure: For the complete analysis this simmulation needs to be completed 2000 times, each time with a different "seed." The current code assumes that each run of the code will be parallelized across a cluster. The "sim" local should be the simulation number, and also dictates the seed and name of the postfile. If a cluster is not available, the entire code (except control structure) could be put into one large loop that iterates through the code 2000 times with `sim' increasing by one each run.
** Primary input: None
** Primary output: 2000 .dta, each with roughly 16 lines of simulation results, each for a different scenario.
** ************************************************************************************************************************

// Control Structure
	// Save (over-write files = 1)
		local save 1

	// Directories
		local DIR "<<Put home directory>>"
		cd `DIR'

	// Local for lauching the simulations via Unix shell file
		local sim `1'

	// Timer
		timer clear
		timer on 1

	// More preferences
		set more off, perm

** ************************************************************************************************************************
```

```
	di `sim'
	set seed `sim'

	// Set up postfile to save results in
		cap postclose simulation`sim'
		if (`save'==1) postfile simulation`sim' J n rho var_x share_var_x_within beta psi pi auto sim beta_re beta_fe yhat_me_re
yhat_mae_re yhat_rmse_re yhat_me_fe yhat_mae_fe yhat_rmse_fe hausman_p ldv_coef r2_LSDV var_x_total var_x_between var_x_within
beta_wb beta_m_wb yhat_me_wb yhat_mae_wb yhat_rmse_wb using "`DIR'/simulation`sim'", replace every(1)

	// Diverse combination of dataset features
		foreach J in 10 50 100 {
		foreach n in 5 10 50 {
		foreach rho in 0 .1 .2 .3 .4 .5 .6 .7 {
		foreach var_x in .5 1 2 {
		foreach share_var_x_within in .1 .25 .5 .75 .9 {
		foreach psi in 0 .2 {
		foreach beta in 1 {
		foreach pi in .1 .25 .5 .75 .9 {
		foreach auto in 0 1 {


	// Create one dataset for each combination of features looped through above
		if (`psi'!=0 | `auto'!=1) {
		di as red "J = `J'; N = `n'; rho = `rho'; var_x = `var_x'; share_var_x_within = `share_var_x_within'; psi = `psi'; beta = `beta'; pi = `pi';
auto = `auto'"

		cap restore
		clear
		clear matrix

		// Set up data structure
```

```
	local N = `J'*`n'
	set obs `N'
	gen N = _n

	gen unit = ceil(N/`n')
	egen year = rank(N), by(unit)
	xtset unit year

// Read input parameters and make necessary variances and standard deviations
	local var_x_w = `share_var_x_within'*`var_x'
	local var_x_b = (1-`share_var_x_within')*`var_x'
	local sigma_x = sqrt(`var_x')
	local sigma_x_w = sqrt(`var_x_w')
	local sigma_x_b = sqrt(`var_x_b')
	local sigma_e = .

// Generate group-means of X and "unobserved" group effects
	local kappa = `rho'*`sigma_x'
	mat temp1 = (1, `kappa' \ `kappa', `var_x_b')

	preserve
	keep unit
	duplicates drop
	cap drawnorm alpha x_mean, cov(temp1)
	tempfile temp`sim'
	save `temp`sim''
	restore
	merge m:1 unit using `temp`sim''
	cap confirm variable alpha
	if (_rc==111) di as red "Failure because temp1 is not positive semi-definite."

// Generate X and residual
```

```
	if (_rc!=111) {
		gen x = rnormal(x_mean, `sigma_x_w')

		local a = 1-(1/`pi')
		local b = (2*`sigma_x'*`psi'*sqrt(1+`var_x'+(2*`rho'*`sigma_x')))/(sqrt(1+`var_x'))
		local c = 1+`var_x'+(2*`rho'*`sigma_x')
		local sigma_e1 = ((-1*`b')+sqrt(`b'^2-4*`a'*`c'))/(2*`a')
		local sigma_e2 = ((-1*`b')-sqrt(`b'^2-4*`a'*`c'))/(2*`a')
		di "Sigma_e1 = `sigma_e1'; sigma_e2 = `sigma_e2'"
		if (`sigma_e1'>`sigma_e2') local sigma_e = `sigma_e1'
		if (`sigma_e1'<=`sigma_e2') local sigma_e = `sigma_e2'

		if (`sigma_e'<=0 | `sigma_e'==.) di as red "Failure because sigma_e < 0"
		if (`sigma_e'>0) {
			mat temp2 = (1, `psi' \ `psi', 1)
			mat temp3 = cholesky(temp2)
			gen temp4 = rnormal(0,`sigma_e')
			if (`auto'==0) gen e = temp3[2,1]*x + temp3[2,2]*temp4
			if (`auto'==1) {
				xtset unit year
				gen e = rnormal(0,`sigma_e') if year==1
				forvalues y = 2(1)`n' {
					qui replace e = temp3[2,1]*l.e + temp3[2,2]*temp4 if year==`y'
					}
				}
// Generate Y
		gen y = alpha + `beta'*x + e

// Test correlations, means, and variances
		sum y alpha x_mean x e
		cor y alpha x_mean x e
		xtserial y x
```

```
// Generate some basic stats
        qui {
        xi: reg y x i.unit
            local r2_LSDV = e(r2)
            local r2_a_LSDV = e(r2_a)
        sum x
            local var_x_total = r(Var)
        sum x_mean
            local var_x_between = r(Var)
        egen sd_x_within = sd(x), by(unit)
        sum sd_x_within, meanonly
            local var_x_within = (r(mean)^2)
        if (`auto'==1) {
            qui reg y l.y x
            local ldv_coef = _b[L1.y]
            }
        else {
            local ldv_coef = .
            }
        }

// Apply RE, FE, and WB estimators and Hausman test
    // RE and FE regressions, and beta errors and prediction errors
        qui {
            foreach method in re fe {
                xtreg y x, `method'
                    local beta_`method' = _b[x]
                estimates store `method'
                predict yhat_`method', xbu
                gen yhat_e_`method' = yhat_`method'-y
                sum yhat_e_`method', meanonly
```

```
                    local yhat_me_`method' = r(mean)
                gen yhat_ae_`method' = abs(yhat_`method'-y)
                sum yhat_ae_`method', meanonly
                    local yhat_mae_`method' = r(mean)
                gen yhat_se_`method' = yhat_e_`method'^2
                sum yhat_se_`method', meanonly
                    local yhat_rmse_`method' = sqrt(r(mean))
                }
            }

// "Within-between" estimator (augmented RE estimation)
    qui {
        gen x_demean = x-x_mean
        xtreg y x_demean x_mean, re
            local beta_wb = _b[x_demean]
            local beta_m_wb = _b[x_mean]
        predict yhat_wb, xbu
        gen yhat_e_wb = yhat_wb-y
        sum yhat_e_wb, meanonly
            local yhat_me_wb = r(mean)
        gen yhat_ae_wb = abs(yhat_wb-y)
        sum yhat_ae_wb, meanonly
            local yhat_mae_wb = r(mean)
        gen yhat_se_wb = yhat_e_wb^2
        sum yhat_se_wb, meanonly
            local yhat_rmse_wb = sqrt(r(mean))
        }

// Hausman test
        qui hausman fe re, sigmamore
        if (r(chi2)>0) local hausman_p = r(p)
        if (r(chi2)<=0) local hausman_p = .
```

```
					}
				}
					if (_rc==111 | `sigma_e'==.) {
						local hausman_p = .
						local ldv_coef = .
						local r2_LSDV = .
						local var_x_total .
						local var_x_between .
						local var_x_within .
						foreach method in re fe wb wbwo {
							local beta_`method' = .
							local yhat_me_`method' = .
							local yhat_mae_`method' = .
							local yhat_rmse_`method' = .
							local beta_m_`method' = .
							}
						}

			// Save estimates in postfile
				if (`save'==1) post simulation`sim' (`J') (`n') (`rho') (`var_x') (`share_var_x_within') (`beta') (`psi') (`pi') (`auto') (`sim') (`beta_re')
(`beta_fe') (`yhat_me_re') (`yhat_mae_re') (`yhat_rmse_re') (`yhat_me_fe') (`yhat_mae_fe') (`yhat_rmse_fe') (`hausman_p') (`ldv_coef')
(`r2_LSDV') (`var_x_total') (`var_x_between') (`var_x_within') (`beta_wb') (`beta_m_wb') (`yhat_me_wb') (`yhat_mae_wb') (`yhat_rmse_wb')
			}
			}
			}
			}
			}
			}
			}
			}
			}
			}
```

```
// Close and save postfile,
	if (`save'==1) postclose simulation`sim'

	timer off 1
	timer list 1

// Open up postfile to examine results across entire set of combinations of features
	use "`DIR'/simulation`sim'.dta", clear
```
