# Supplementary material for: Correction: Random-Effects, Fixed-Effects and the within-between Specification for Clustered Data in Observational Health Studies: A Simulation Study
Source: PLoS One. 2016 May 24;11(5):e0156508. doi: 10.1371/journal.pone.0156508 (PMC4878732; doi:10.1371/journal.pone.0156508)
Supplement: S1 File — (PDF) [file pone.0156508.s001.pdf]

```
** ****************************************************************************************************************
** Authors: Joe Dieleman and Tara Templin
** File Created: March 2013
** Updated: August 2014, March 2016
** Objective: Simulate one dataset for each combination of features, apply RE,FE, WB estimation and the Hausman test to each dataset, and save results in a postfile.
** Structure: For the complete analysis this simulation needs to be completed 2000 times, each time with a different "seed." The current code assumes that each run of the code will be parallelized across a cluster. The "sim" local should be the simulation number, and also dictates the seed and name of the postfile. If a cluster is not available, the entire code (except control structure) could be put into one large loop that iterates through the code 2000 times with `sim' increases by one each run.
** Primary input: None
** Primary output: 2000 .dta, each with roughly 16 lines of simulation results, each for a different scenario.
** ****************************************************************************************************************

** ****************************************************************************************************************
// Control Structure
	// Save (over-write files = 1)
		local save 1

	// Directories
		local DIR "<<put working directory here>>"
		cd `DIR'

	// Local for launching the simulations via Unix shell file
		local sim `1'

	// Timer
		timer clear
		timer on 1

	// More preferences
		set more off, perm

** ****************************************************************************************************************

	di `sim'
	set seed `sim'

	// Set up postfile to save results in
		cap postclose simulation`sim'
		if (`save'==1) postfile simulation`sim' J n rho var_x share_var_x_within beta psi pi auto sim beta_re beta_fe yhat_me_re yhat_mae_re yhat_rmse_re yhat_me_fe yhat_mae_fe yhat_rmse_fe hausman_p ldv_coef r2_LSDV var_x_total var_x_between var_x_within beta_wb beta_m_wb yhat_me_wb yhat_mae_wb yhat_rmse_wb using "`DIR'/simulation`sim'", replace every(1)

	// Diverse combination of dataset features
		foreach J in 10 50 100 {
		foreach n in 5 10 50 {
		foreach rho in 0 .1 .2 .3 .4 .5 .6 .7 {
		foreach var_x in .5 1 2 {
		foreach share_var_x_within in .1 .25 .5 .75 .9 {
		foreach psi in 0 .2 {
		foreach beta in 1 {
		foreach pi in .1 .25 .5 .75 .9 {
		foreach auto in 0 1 {

	// Create one dataset for each combination of features looped through above
		if (`psi'!=0 | `auto'!=1) {
```

```
di as red "J = `J'; N = `n'; rho = `rho'; var_x = `var_x'; share_var_x_within = `share_var_x_within'; psi = `psi'; beta = `beta'; pi = `pi'; auto = `auto'"

cap restore
clear
clear matrix

// Set up data structure
	local N = `J'*`n'
	set obs `N'
	gen N = _n

	gen unit = ceil(N/`n')
	egen year = rank(N), by(unit)
	xtset unit year

// Read input parameters and make necessary variances and standard deviations
	local var_x_w = `share_var_x_within'*`var_x'
	local var_x_b = (1-`share_var_x_within')*`var_x'
	local sigma_x = sqrt(`var_x')
	local sigma_x_w = sqrt(`var_x_w')
	local sigma_x_b = sqrt(`var_x_b')
	local sigma_e = .

// Generate group-means of X and "unobserved" group effects
	local kappa = `rho'*`sigma_x'
	mat temp1 = (1, `kappa' \ `kappa', `var_x_b')

	preserve
	keep unit
	duplicates drop
	cap drawnorm alpha x_mean, cov(temp1)
	tempfile temp`sim'
	save `temp`sim''
	restore
	merge m:1 unit using `temp`sim''
	cap confirm variable alpha
	if (_rc==111) di as red "Failure because temp1 is not positive semi-definite."

// Generate X and residual
	if (_rc!=111) {
		gen x = rnormal(x_mean, `sigma_x_w')
		egen x_mean_obs = mean(x), by(unit)
		local a = 1-(1/`pi')
		local b = (2*`sigma_x'*`psi'*sqrt(1+`var_x'+(2*`rho'*`sigma_x')))/(sqrt(1+`var_x'))
		local c = 1+`var_x'+(2*`rho'*`sigma_x')
		local sigma_e1 = ((-1*`b')+sqrt(`b'^2-4*`a'*`c'))/(2*`a')
		local sigma_e2 = ((-1*`b')-sqrt(`b'^2-4*`a'*`c'))/(2*`a')
		di "Sigma_e1 = `sigma_e1'; sigma_e2 = `sigma_e2'"
		if (`sigma_e1'>`sigma_e2') local sigma_e = `sigma_e1'
		if (`sigma_e1'<=`sigma_e2') local sigma_e = `sigma_e2'

		if (`sigma_e'<=0 | `sigma_e'==.) di as red "Failure because sigma_e < 0"
		if (`sigma_e'>0) {
			mat temp2 = (1, `psi' \ `psi', 1)
```

```
						mat temp3 = cholesky(temp2)
						gen temp4 = rnormal(0,`sigma_e')
						if (`auto'==0) gen e = temp3[2,1]*x + temp3[2,2]*temp4
						if (`auto'==1) {
							xtset unit year
							gen e = rnormal(0,`sigma_e') if year==1
							forvalues y = 2(1)`n' {
								qui replace e = temp3[2,1]*l.e + temp3[2,2]*temp4 if year==`y'
								}
							}
		// Generate Y
				gen y = alpha + `beta'*x + e

		// Test correlations, means, and variances
				sum y alpha x_mean x e
				cor y alpha x_mean x e
				** xtserial y x

		// Generate some basic stats
				qui {
				xi: reg y x i.unit
					local r2_LSDV = e(r2)
					local r2_a_LSDV = e(r2_a)
				sum x
					local var_x_total = r(Var)
				sum x_mean
					local var_x_between = r(Var)
				egen sd_x_within = sd(x), by(unit)
				sum sd_x_within, meanonly
					local var_x_within = (r(mean)^2)
				if (`auto'==1) {
					qui reg y l.y x
					local ldv_coef = _b[L1.y]
					}
				else {
					local ldv_coef = .
					}
				}


	// Apply RE, FE, and WB estimators and Hausman test
		// RE and FE regressions, and beta errors and prediction errors
				qui {
					foreach method in re fe {
						xtreg y x, `method'
							local beta_`method' = _b[x]
						estimates store `method'
						predict yhat_`method', xbu
						gen yhat_e_`method' = yhat_`method'-y
						sum yhat_e_`method', meanonly
							local yhat_me_`method' = r(mean)
						gen yhat_ae_`method' = abs(yhat_`method'-y)
						sum yhat_ae_`method', meanonly
							local yhat_mae_`method' = r(mean)
```

```
				gen yhat_se_`method' = yhat_e_`method'^2
				sum yhat_se_`method', meanonly
					local yhat_rmse_`method' = sqrt(r(mean))
				}
			}

	// "Within-between" estimator (augmented RE estimation)
		qui {
			gen x_demean = x-x_mean_obs
			xtreg y x_demean x_mean_obs, re
				local beta_wb = _b[x_demean]
				local beta_m_wb = _b[x_mean_obs]
			predict yhat_wb, xbu
			gen yhat_e_wb = yhat_wb-y
			sum yhat_e_wb, meanonly
				local yhat_me_wb = r(mean)
			gen yhat_ae_wb = abs(yhat_wb-y)
			sum yhat_ae_wb, meanonly
				local yhat_mae_wb = r(mean)
			gen yhat_se_wb = yhat_e_wb^2
			sum yhat_se_wb, meanonly
				local yhat_rmse_wb = sqrt(r(mean))
			}

	// Hausman test
			qui hausman fe re, sigmamore
			if (r(chi2)>0) local hausman_p = r(p)
			if (r(chi2)<=0) local hausman_p = .
		}
	}
		if (_rc==111 | `sigma_e'==.) {
			local hausman_p = .
			local ldv_coef = .
			local r2_LSDV = .
			local var_x_total .
			local var_x_between .
			local var_x_within .
			foreach method in re fe wb wbwo {
				local beta_`method' = .
				local yhat_me_`method' = .
				local yhat_mae_`method' = .
				local yhat_rmse_`method' = .
				local beta_m_`method' = .
				}
			}

// Save estiamtes in postfile
	if (`save'==1) post simulation`sim' (`J') (`n') (`rho') (`var_x') (`share_var_x_within') (`beta') (`psi') (`pi') (`auto') (`sim') (`beta_re') (`beta_fe') (`yhat_me_re') (`yhat_mae_re')
(`yhat_rmse_re') (`yhat_me_fe') (`yhat_mae_fe') (`yhat_rmse_fe') (`hausman_p') (`ldv_coef') (`r2_LSDV') (`var_x_total') (`var_x_between') (`var_x_within') (`beta_wb') (`beta_m_wb') (`yhat_me_wb')
(`yhat_mae_wb') (`yhat_rmse_wb')
}
}
}
}
```

```
		}
		}
		}
		}
		}
		}

// Close and save postfile,
		if (`save'==1) postclose simulation`sim'

		timer off 1
		timer list 1

// Open up postfile to examine results across entire set of combinations of features
		use "`DIR'/simulation`sim'.dta", clear
```
